# A Previous Miscarriage and a Previous Successful Pregnancy Have a Different Impact on HLA Antibody Formation during a Subsequent Successful Pregnancy

**DOI:** 10.3389/fimmu.2016.00571

**Published:** 2016-12-06

**Authors:** Kirsten Geneugelijk, Gideon Hönger, Hanneke Wilhelmina Maria van Deutekom, Irene Mathilde Hösli, Stefan Schaub, Eric Spierings

**Affiliations:** ^1^Laboratory for Translational Immunology, University Medical Center Utrecht, Utrecht, Netherlands; ^2^Laboratory for Transplantation Immunology and Nephrology, Department of Biomedicine, University Hospital Basel, Basel, Switzerland; ^3^Department of Theoretical Biology and Bioinformatics, University Utrecht, Utrecht, Netherlands; ^4^Department for Obstetrics and Fetomaternal Medicine, University Hospital Basel, Basel, Switzerland; ^5^Clinic for Transplantation Immunology and Nephrology, University Hospital Basel, Basel, Switzerland

**Keywords:** allo-sensitization, HLA antibodies, pregnancy, miscarriage, PIRCHE-II

## Abstract

Inherited paternal HLA antigens from the semi-allogeneic fetus may trigger maternal immune responses during pregnancy, leading to the production of child-specific HLA antibodies. The prevalence of these HLA antibodies increases with the number of successful pregnancies. In the present study, we investigated the effect of a single prior miscarriage on HLA antibody formation during a subsequent successful pregnancy. Women with a successful pregnancy with one or more prior miscarriages (*n* = 229) and women with a successful pregnancy without a prior miscarriage (*n* = 58), and their children were HLA typed. HLA antibody analyses were performed in these women to identify whether HLA antibodies were formed against mismatched HLA class-I antigens of the last child. The percentage of immunogenic antigens was significantly lower after a single successful pregnancy that was preceded by a single miscarriage (*n* = 18 women) compared to a successful pregnancy that was preceded by a first successful pregnancy (*n* = 62 women). Thus, our data suggest that a previous miscarriage has a different impact on child-specific HLA antibody formation during a subsequent successful pregnancy than a previous successful pregnancy. The lower immunogenicity in these women cannot be explained by reduced numbers of immunogenic B-cell and T-cell epitopes. In conclusion, our observations indicate that increasing gravidity is not related to an increased prevalence of HLA antibodies in a single successful pregnancy that was preceded by a single prior miscarriage.

## Introduction

A successful pregnancy requires an optimal interplay between the maternal immune system and the semi-allogeneic fetus. Breakdown of the maternal immune tolerance may result in fetal rejection. Thus, the maternal tolerance toward the fetus has to be maintained both locally at the fetal-maternal interface and systemically, since bidirectional trafficking of cells and soluble HLA between the mother and the fetus takes place (1–3). As early as 4 weeks of gestation, semi-allogeneic fetal DNA can be detected in the maternal circulation (2) and the presence of this fetal microchimerism can persist for decades after delivery (4).

Inherited paternal HLA antigens (IPA) of fetal origin are able to prime maternal immune responses at the fetal-maternal interface as well as in the maternal circulation (5, 6). These immune responses may lead to the production of child-specific HLA antibodies (7–9). The maternal production child-specific HLA antibodies of the IgG isotype requires interaction between activated B-cells and primed T-helper cells. First, B-cell activation occurs upon antigenic uptake of IPA by the B-cell receptor (10). Subsequently, upon T-cell recognition of degraded IPA presented on HLA class-II molecules, T-helper cells provide co-stimulation *via* CD40–CD40L interaction and secrete cytokines (10, 11). These signals drive proliferation and differentiation of naive B cells into memory cells and plasma cells and induce IgM to IgG isotype switching (10, 11). Thus, the maternal production of child-specific IgG HLA antibodies requires the activation of B cells by T-helper cells where both B cells and T-helper cells respond to the same antigen, a phenomenon called linked recognition (12).

Despite abundant allogeneic fetal contact, only 10–40% of the mothers develop child-specific HLA antibodies (8, 9). The exact mechanism behind HLA antibody formation is currently unclear. Increasing gravidity (8, 13) and the fetal and maternal HLA phenotype combination (14) may be important determinants in the immunogenicity toward IPA. We previously showed that HLA antibody formation during a successful pregnancy without prior miscarriages is related to the number of predicted HLA-derived T-helper epitopes as determined by the PIRCHE-II model (Predicted Indirectly ReCognizable HLA Epitopes) (15). This model identifies the number of mismatched HLA-derived peptides that can be presented by HLA class-II molecules, designated as PIRCHE-II (16).

HLA antibodies play an important role in organ transplantation; the presence of pre-transplantation donor-specific HLA antibodies is associated with antibody-mediated rejection and an impaired graft survival (17–20). Therefore, more insight into the immunogenicity of mismatched HLA after pregnancy may have has implications in the transplantation field. In contrast to transplantation, the effect of IPA-specific HLA antibodies on the fetus is presumably rather harmless, as the prevalence of IPA-specific HLA antibodies is relatively high in normal pregnancies. However, both beneficial and harmful effects of HLA antibodies on pregnancy outcome have been described, indicating that the role of IPA-specific HLA antibodies on pregnancy outcome is debatable (21). Most of these studies focused on HLA antibody formation in (recurrent) miscarriage(s), whereas studies about the effect of a prior miscarriage on HLA antibody formation during a subsequent successful pregnancy are limited. In the present study, we investigate for the first time the effect of a single previous miscarriage on HLA antibody formation during a subsequent first successful pregnancy.

## Materials and Methods

### Population and Sample Collection

We included in this study 301 mothers who gave birth between September 2009 and April 2011 at the University Hospital Basel, Switzerland. All women included had either their first full-term pregnancy or gave birth to children from the same partner before. Fully HLA class-I matched mother–child pairs (*n* = 3) were excluded from the analyses. In some mother–child pairs, the child was homozygous for a HLA class-I IPA for which the mother was heterozygous (*n* = 8). These mother–child pairs were also excluded from analyses, as these HLA class-I IPA was identical to the mother and thus not immunogenic. From all participating women, blood transfusions and previous miscarriages were documented. Three women had previous blood transfusions, and these mother–child pairs were excluded from further analysis. From the remaining 287 mother–child pairs, a total of 58 women had one or more prior miscarriages. These women with one of more prior miscarriages were used to study the effect of a prior miscarriage on HLA antibody formation during a subsequent successful pregnancy.

After obtaining informed consent from all the participating women, blood samples were taken from the mother 1–4 days after delivery. Cord blood of the child was sampled directly after delivery. HLA antibody analysis was performed on the maternal blood samples, and HLA typing was performed on blood samples that were obtained from both the mother and the cord blood. This study was approved by the local ethics committee (EKBB; reference number 23/09).

### HLA Typing

High-resolution HLA typing was performed on maternal blood samples and cord blood samples using either sequence-based typing (www.histogenetics.com) or SSO DNA typing (LABType HD; One Lambda). Identification of mismatched IPA was based on two-field resolution HLA typing of both mother and child.

### HLA Antibody Analysis

Maternal post-delivery blood samples were analyzed for the presence of HLA antibodies using single HLA class I-antigen beads according to the instructions of the manufacturer (iBeads Lot 1; One Lambda) as described previously (9). For the analyses presented in this paper, we consider mean fluorescence intensity >1,000 as positive. Mismatched HLA class-I IPA against which the mother had developed HLA-specific antibodies were classified as immunogenic HLA, whereas mismatched HLA class-I IPA against which the mother had not developed HLA-specific antibodies were classified as non-immunogenic HLA. The percentage of immunogenic antigens was calculated for individual groups by dividing the number of immunogenic HLA by the total number of HLA class-I IPA mismatches multiplied by 100%.

### Identification of HLA Class-I-Derived PIRCHE-II

The numbers of HLA class-I derived epitopes from the child presented by maternal HLA class-II molecules, PIRCHE-II, were determined as described previously (15). Briefly, for all mismatched HLA-A, HLA-B, and HLA-C antigens of the child, we used the netMHCIIpan-3.0 algorithm to predict how mismatched HLA-derived peptide may align in the binding groove of maternal HLA-DRB1 [algorithm available *via* http://www.cbs.dtu.dk/services/NetMHCIIpan-3.0/ (22)]. Subsequently, the binding affinity of this peptide to maternal HLA-DRB1 was predicted by the algorithm, considering binding affinities with an IC_50_ of <1,000 nM as relevant HLA-DRB1 binders. HLA-DRB1 binders were designated as a PIRCHE-II when the predicted binders differed at least one amino acid with the maternal HLA amino acid sequence. Only unique child-specific epitope-HLA complexes were counted as a PIRCHE-II. The PIRCHE algorithm is available *via* http://www.pirche.org.

### HLAMatchmaker

HLAMatchmaker version 2.1 was used to determine the number of HLAMatchmaker eplets for all mismatched HLA class-I molecules of the child. Eplets that were present in HLA of the child and absent in the mother’s HLA-A, HLA-B, HLA-C, and HLA-DRB1 locus were counted as mismatched eplets. The HLAMatchmaker software is available *via* http://www.epitopes.net (23).

### Statistical Analysis

We used the GraphPad Prism software version 6.02 (GraphPad Software, Inc., La Jolla, CA, USA) and the SPSS Statistics software version 20 (IBM SPSS Software) for the statistical analyses. Pearson’s chi-square tests were used to analyze differences in percentage of immunogenic antigens between different groups. Mann–Whitney *U* tests were used to analyze differences in the number of mismatched eplets and PIRCHE-II between different groups. *p*-values <0.05 were assumed to indicate statistical significance.

## Results

### Population Characteristics

Table 1 summarizes the characteristics of the study population. Of all 287 women, the majority of the women (79.8%) did not have any prior miscarriage. A total of 58 women had one or more prior miscarriages. The majority of these women with a prior miscarriage had a single prior miscarriage. In all 287 women, 738 HLA-class I IPA mismatches were identified. Table 2 summarizes the number of mismatched IPA for pregnancies with and without prior miscarriage(s) and the percentage of immunogenic HLA per locus. The percentage of immunogenic IPA between these groups did not significantly differ (*p* = 0.72, *p* = 0.64, and *p* = 0.08 for HLA-A, HLA-B, and HLA-C, respectively, in Pearson’s chi-square tests with Yates’ correction).

**Table 1:** **Population characteristics**.

	Without prior miscarriage, *n* (%)	With prior miscarriage(s), *n* (%)		
		1 prior miscarriage	2 prior miscarriages	≥3 prior miscarriages
First full-term pregnancy	154 (53.7)	18 (6.3)	7 (2.4)	4 (1.4)
Second full-term pregnancy	65 (22.6)	15 (5.2)	5 (1.7)	2 (0.7)
Third or more full-term pregnancy	10 (3.5)	4 (1.4)	2 (0.7)	1 (0.3)

**Table 2:** **Number of mismatched inherited paternal HLA antigens (IPA) per locus; *n* (% immunogenic IPA per locus)**.

	HLA-A	HLA-B	HLA-C
Pregnancies without prior miscarriage	234 (16%)	259 (17%)	245 (6%)
Pregnancies with prior miscarriage(s)	40 (20%)	54 (20%)	48 (15%)

### First Pregnancy and First Miscarriage Have a Different Impact on HLA Antibody Formation during a Subsequent Successful Pregnancy

**Figure 1:**
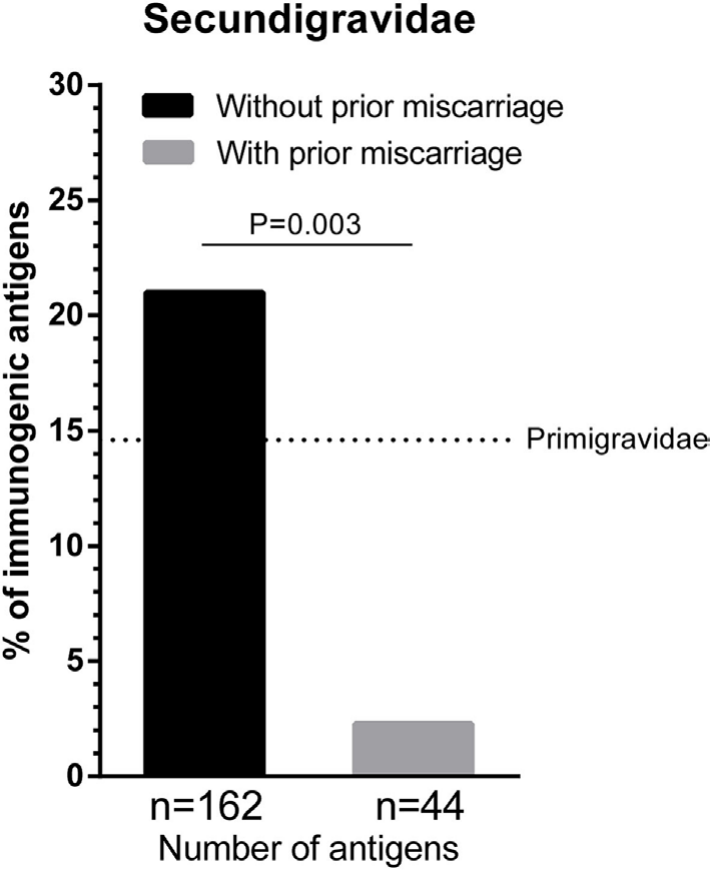
**The effect of first pregnancy and first miscarriage on subsequent successful pregnancy**. The percentage of immunogenic antigens is higher for secundigravidae without a prior miscarriage (black bar) than secundigravidae with a prior miscarriage (gray bar). The dotted line represents the percentage of immunogenic antigens for primigravidae (women with a single successful full-term pregnancy; 14.6%). For each group, *n* represents the number of mismatched antigens. The *p* value is derived from Pearson’s chi-square test.

Multiple successful pregnancies and prior miscarriages may have a differential effect on HLA immunization during a subsequent successful pregnancy. To investigate the effect of a first pregnancy and a first miscarriage on HLA antibody formation during a subsequent successful pregnancy, we compared secundigravidae without a prior miscarriage (i.e., these women had two successful pregnancies without a prior miscarriage; *n* = 65 women) with secundigravidae with a prior miscarriage (i.e., these women had a single successful pregnancy that was preceded by a single miscarriage; *n* = 18 women) (Figure 1). The secundigravidae without a prior miscarriage group had a total of 162 HLA class I mismatched IPA, whereas the secundigravidae with a prior miscarriage had 44 HLA class I-mismatched IPA. The percentage of immunogenic antigens was higher for secundigravidae without a prior miscarriage (21%) compared to secundigravidae with a prior miscarriage (2.3%) (Figure 1; *p* = 0.003). For the secundigravidae with a prior miscarriage, only a single HLA was immunogenic (HLA-C*01:02), while the other 43 mismatched HLA were non-immunogenic. When using a lower fluorescence intensity cutoff (>500), the percentage of immunogenic antigens for secundigravidae with a prior miscarriage increased marginally (4.5%). These observations indicate that the HLA immunogenicity is significantly lower during a subsequent successful pregnancy in women who experienced a prior miscarriage compared to women who had a prior successful pregnancy. The percentage of immunogenic antigens in the secundigravidae with a prior miscarriage group was also lower than the percentage of immunogenic antigens in the primigravidae group (i.e., these women had a single successful pregnancy without a history of prior miscarriages) (dotted line in Figure 1; 14.6%; primigravidae versus secundigravidae with a prior miscarriage: *p* = 0.02), indicating that the immunization pattern observed in secundigravidae with a prior miscarriage is not similar to the immunization pattern observed in primigravidae.

**Figure 2:**
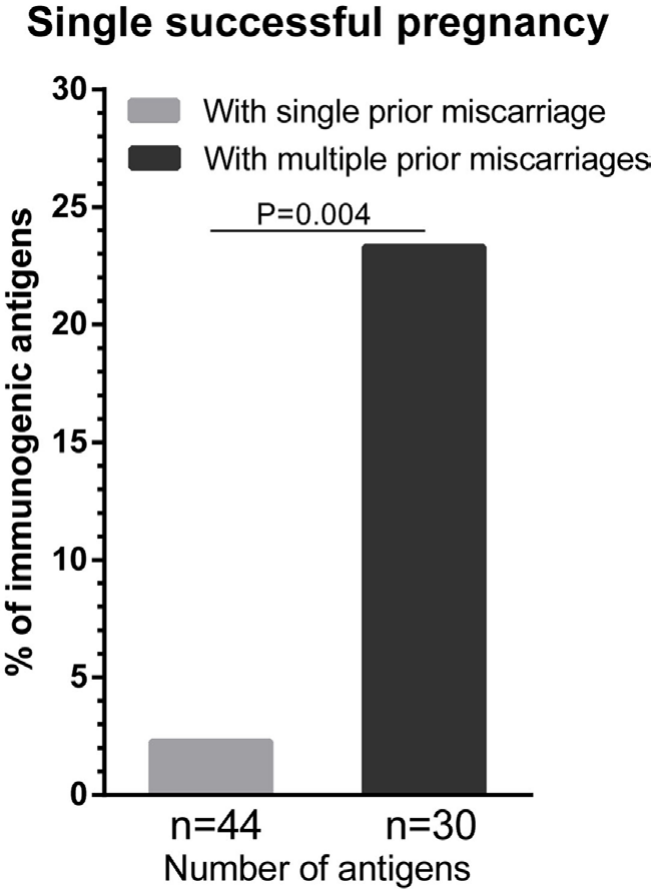
**The effect of the number of prior miscarriage on HLA sensitization during a subsequent successful pregnancy**. HLA sensitization in women with a single successful pregnancy that was preceded by a single prior miscarriage was compared with HLA sensitization in women with a single successful pregnancy that was preceded by multiple prior miscarriages. The percentage of immunogenic antigens is higher for women with multiple a prior miscarriages (dark gray bar) than for women with a single prior miscarriage (light gray bar). For each group, *n* represents the number of mismatched antigens. The *p* value is derived from Pearson’s chi-square test.

Next, we investigated the effect of the number of prior miscarriages on HLA sensitization during a subsequent successful pregnancy. We compared the percentage of immunogenic antigens between women with a single successful pregnancy that was preceded by a single prior miscarriage (i.e., secundigravidae with a prior miscarriage) and women with a single successful pregnancy that was preceded by multiple prior miscarriages (Figure 2). For women with multiple prior miscarriages, the percentage of immunogenic HLA was higher (23.3%) compared to women with a single prior miscarriage (2.3%) (Figure 2; *p* = 0.004), indicating that the number of prior miscarriages may influence HLA sensitization during a subsequent successful pregnancy.

**Figure 3:**
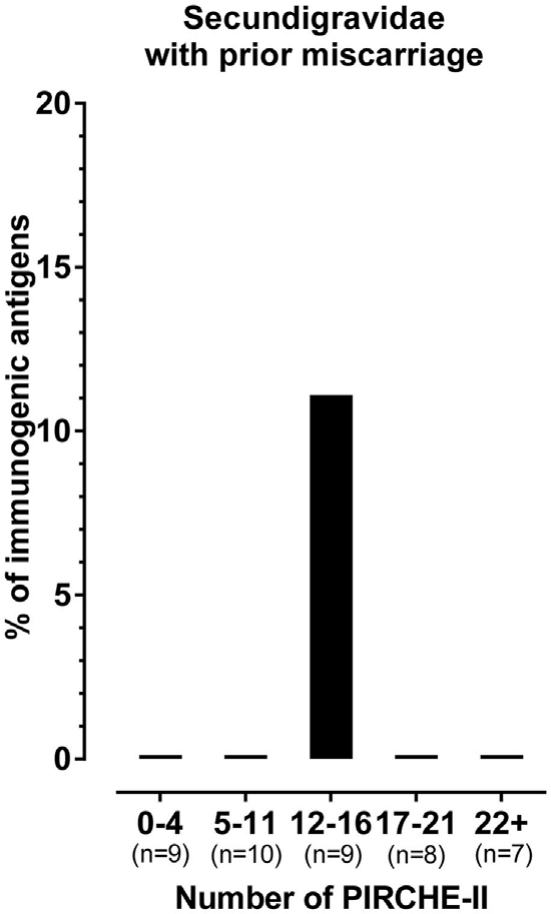
**The single immunogenic HLA in the secundigravidae with a prior miscarriage group has a median number of PIRCHE-II**. The number of PIRCHE-II was divided into quintiles (0–4 PIRCHE-II, 5–11 PIRCHE-II, 12–16 PIRCHE-II, 17–21 PIRCHE-II, and 22+ PIRCHE-II). For each individual quintile, the percentage of immunogenic antigens was plotted. All HLA mismatches present in the 0–4 PIRCHE-II, 5–11 PIRCHE-II, 17–21 PIRCHE-II, and the 22+ PIRCHE-II quintiles were non-immunogenic, resulting in 0% immunogenic antigens. The single immunogenic HLA-C*01:02 is present in the 12–16 PIRCHE-II quintile, whereas the other HLA mismatches present in this PIRCHE-II quintile were non-immunogenic, resulting in 11.1% immunogenic antigens. For each group, *n* represents the number of mismatched antigens.

We previously showed that the probability of HLA antibody formation increases with the number of PIRCHE-II in successful pregnancies without a prior miscarriage (15). Thus, we showed that in these pregnancies, including secundigravidae without a prior miscarriage, a higher number of PIRCHE-II was related to a higher percentage of immunogenic antigens. Therefore, one could hypothesize that the single immunogenic HLA-C*01:02 in the secundigravidae with prior miscarriage group has a higher number of PIRCHE-II compared to the other non-immunogenic HLA. To investigate this aspect in the secundigravidae with a prior miscarriage group, the PIRCHE-II numbers for the mismatched antigens were divided into quintiles (i.e., five equal groups) (Figure 3). For each of these quintiles, we plotted the percentage of immunogenic antigens, and we investigated in which quintile the single immunogenic HLA-C*01:02 of secundigravidae with a prior miscarriage was present. The single immunogenic HLA-C*01:02 is not an outlier, as it was present in the central quintile (12–16 PIRCHE-II). This observation indicates that the lower percentage of immunogenic HLA in the secundigravidae with a prior miscarriage group cannot be explained by having an increased or a reduced number of PIRCHE-II compared to non-immunogenic HLA.

### The Lower Immunogenicity in Secundigravidae with a Prior Miscarriage Is Likely Not Due to Lower Numbers of Immunogenic B Cell and T-Helper Cell Epitopes

**Figure 4:**
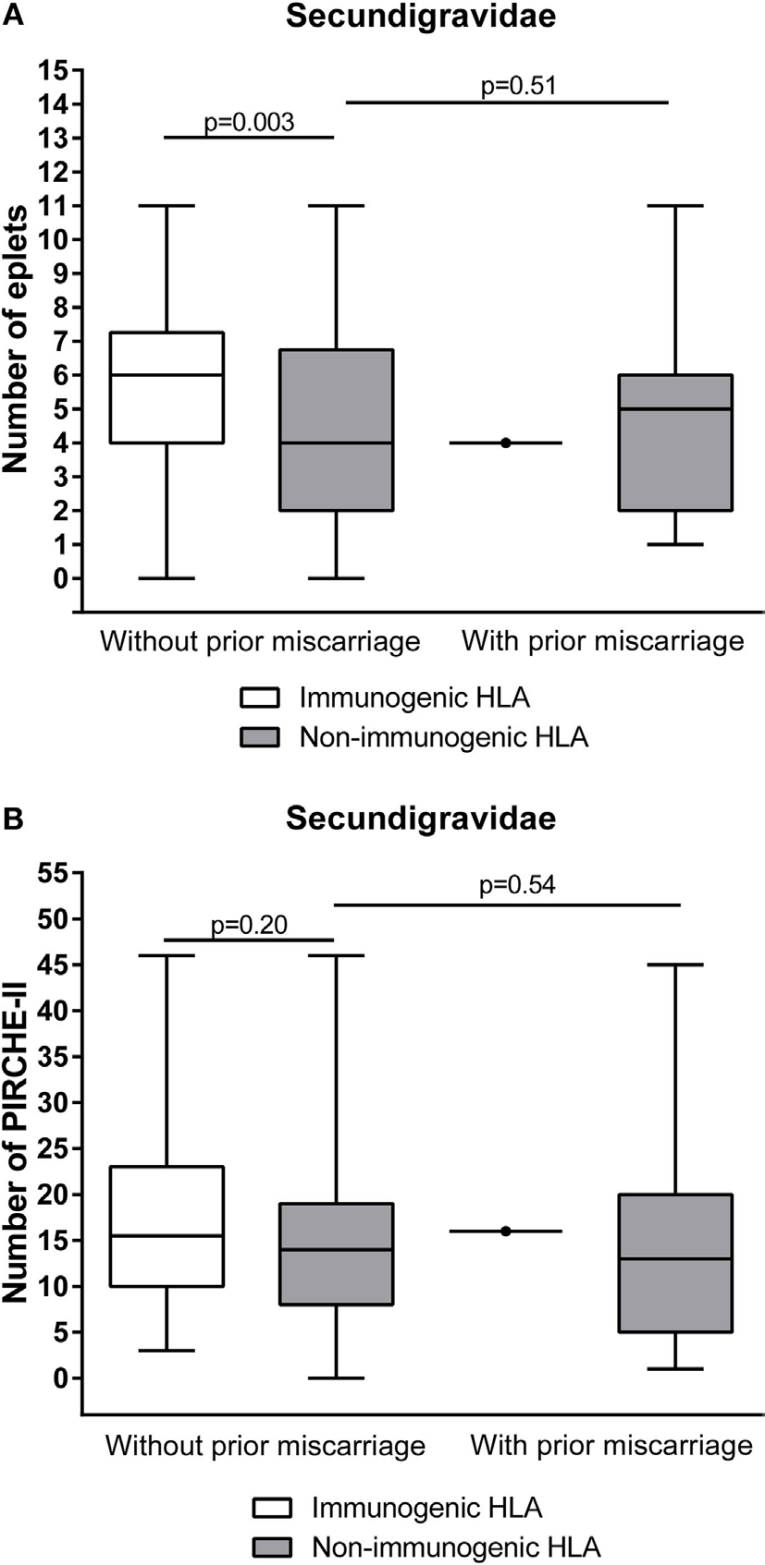
**Comparison of the number of immunogenic factors between secundigravidae without a prior miscarriage and secundigravidae with a prior miscarriage**. Non-immunogenic HLA of secundigravidae with a prior miscarriage contains a similar number of mismatched eplets **(A)** and PIRCHE-II **(B)** compared to non-immunogenic HLA of secundigravidae without a prior miscarriage. For the secundigravidae with a prior miscarriage group, the single immunogenic HLA is depicted as a dot. The reported *p*-values are derived from Mann–Whitney *U* tests. The boxes extend from the 25th to 75th percentiles, and the middle line represents the median. The whiskers are drawn from the lowest to the highest PIRCHE-II value.

The ability to develop HLA antibodies against child-specific HLA mismatches is determined by allo-epitopes that are present on mismatched HLA. The HLAMatchmaker algorithm identifies the number of antibody-accessible allo-epitopes (eplets) on mismatched HLA that are not present on self-HLA. To investigate whether the lower immunogenicity in secundigravidae with a prior miscarriage is due to a lower number of immunogenic B-cell epitopes in this population, we calculated the number of mismatched eplets for secundigravidae with a prior miscarriage and for secundigravidae without a prior miscarriage (Figure 4A). Since only a single HLA of the secundigravidae with a prior miscarriage is immunogenic, analyses were performed on the non-immunogenic HLA groups of both populations. The number of eplets did not differ between non-immunogenic HLA of secundigravidae with a prior miscarriage and non-immunogenic HLA of secundigravidae without a prior miscarriage (*p* = 0.51). When analyzing the number of PIRCHE-II (T-helper cell epitopes) in both groups (Figure 4B), the number of PIRCHE-II was similar for non-immunogenic HLA of secundigravidae with a prior miscarriage compared to non-immunogenic HLA of secundigravidae without a prior miscarriage (*p* = 0.54). Thus both the eplet and PIRCHE-II numbers are comparable between secundigravidae with a miscarriage and secundigravidae without a miscarriage, indicating that the number of immunogenic factors (i.e., B-cell and T-helper cell epitopes) is not altered in secundigravidae with a prior miscarriage.

## Discussion

Maternal immune responses can be formed against IPA of the fetus during pregnancy, leading to IPA-specific antibodies and T cells (5, 7). Despite the clinical relevance of HLA-specific antibodies in transplantation outcome, the clinical relevance of paternal HLA-specific antibodies in pregnancy outcome is currently unclear (21). The present study was initiated to investigate the effect of a first pregnancy and a first miscarriage on HLA antibody formation during a subsequent first successful pregnancy.

In our cohort of 287 mother–child pairs, we investigated HLA immunization against mismatched IPA of the most recent child in secundigravidae with or without a single prior miscarriage. The percentage of immunogenic HLA was significantly lower in secundigravidae with a prior miscarriage compared to secundigravidae without a prior miscarriage. Several studies have shown that the prevalence of HLA antibodies increases with the number of successful pregnancies (9, 13). Our data show that the relation between increasing gravidity and the prevalence of HLA antibody formation is absent in secundigravidae with a prior miscarriage, indicating that a previous miscarriage behaves differently when compared to a previous successful pregnancy.

Our results suggest that a prior miscarriage has a different immunological impact on a subsequent successful pregnancy than a prior successful pregnancy. The lower immunogenicity observed in secundigravidae with a prior miscarriage cannot be explained by altered numbers of mismatched eplets and PIRCHE-II (Figure 4). Alternatively, the lower percentage of immunogenic antigens among secundigravidae with prior miscarriage in our population may result from tolerizing effects of a first short allogeneic interaction during the prior miscarriage. These tolerizing effects may be caused by fetal microchimerism, as the increased occurrence and long-term persistence of fetal microchimerism in the maternal system after or during fetal loss has been described previously (24, 25). Alternatively, the low percentage of immunogenic antigens among secundigravidae with a single prior miscarriage might also be explained by natural selection of a particular HLA genotype during a subsequent pregnancy. The chance of inheriting an alternative paternal haplotype during a subsequent successful pregnancy compared to the previous miscarriage is 50%. However, a previous miscarriage may further stimulate HLA genotype diversity by putting additive selective pressure on a subsequent pregnancy. Either directly or *via* modulating the maternal immune system, the HLA genotype of the miscarried fetus may discriminate against that particular HLA genotype during or shortly after conception (26). If this hypothesis is correct, a previous miscarried fetus facilitates the selection of the HLA genotype of a subsequent child. Such a selection may be achieved *via* a maternal immune response directed against the HLA genotype that is similar to the HLA genotype of the miscarriage itself, resulting in either selective abortion of the fetus or *via* a female alloimmune response against certain HLA genotypes present in seminal fluid, as seminal plasma contains soluble HLA (27) and spermatozoa also express both HLA class-I and class-II (28). However, currently no data are available to support such a natural selection of a particular HLA genotype. To challenge this hypothesis, the HLA typing of the current child should be compared with the HLA typing of the previous miscarried fetus. HLA typing of the miscarried fetus is not available for the current cohort and is in general hard to obtain. Alternatively, inclusion of paternal HLA typing may provide a better insight in this mechanism.

The duration of maternal exposure to allo-epitopes is significantly shorter during a miscarriage compared to a full-term pregnancy. Therefore, one might argue that alloimmunization is negligible in pregnancies that end in a miscarriage and that the alloimmunization pattern of secundigravidae with a prior miscarriage is more comparable to the alloimmunization pattern of primigravidae. In this study, we showed that the percentage of immunogenic antigens for secundigravidae with a prior miscarriage was also lower than the percentage of immunogenic antigens observed for primigravidae (Figure 1), demonstrating that the immunization pattern in secundigravidae with a prior miscarriage differs from the immunization pattern that was observed in primigravidae. Thus, despite a shorter duration of maternal allo-exposure during pregnancy loss, the effect of a prior miscarriage on a subsequent pregnancy cannot be neglected in terms of HLA antibody formation.

Although our investigation on the differential effect of a first pregnancy and a first miscarriage on a subsequent successful pregnancy are unprecedented, our observation might be supported by previous reports. For example, Triulzi et al. showed that women with a single pregnancy that ended in a miscarriage had a diminished HLA alloimmunization compared to women with a single pregnancy that ended in a successful delivery (13). Furthermore, Masson et al. reported that the HLA immunization incidence was diminished in women with a single successful pregnancy that was preceded by one or more miscarriages compared to women with a single successful pregnancy that was not preceded by one or more miscarriages (29). However, the latter study did not take the number of prior miscarriages into account. In our population, we observed that women with a single successful pregnancy that was preceded by two or more miscarriages had a higher percentage of immunogenic antigens than women with a single successful pregnancy that was preceded by a single miscarriage (Figure 2). This observation indicates that the number of prior miscarriages may have impact on HLA sensitization during a subsequent successful pregnancy.

The probability of HLA antibody formation increases with the number of PIRCHE-II in pregnancies that were not preceded by one or more miscarriages, including secundigravidae without a prior miscarriage (15). In our cohort of secundigravidae with a prior miscarriage, the single immunogenic HLA had a number of PIRCHE-II that was comparable to the other non-immunogenic HLA (Figure 4), indicating that the PIRCHE-II effect is absent in pregnancies that were preceded by miscarriages.

Our study has limited details about the miscarried fetus itself, as the paternity, HLA typing, and cause of the miscarriage were not documented. Furthermore, the miscarriages in our cohort were self-reported. Since a majority of the miscarriages are unnoticed (30), it may well be that the number of prior miscarriages is underestimated. Therefore, also in the secundigravidae without prior miscarriage group and in the primigravidae group some women might have previous miscarriages, which may led to underestimation of immunization toward IPA in normal pregnancies. Moreover, serum samples for HLA antibody analysis after the miscarriage are lacking for our cohort. These latter serum samples may answer the question whether the mother had developed HLA antibodies against the miscarried fetus or not and would provide more insight in the possible mechanisms behind our observations.

In summary, we showed that a previous miscarriage and a previous successful pregnancy have a different impact on HLA antibody formation during a subsequent successful pregnancy. In contrast to successful pregnancies, increasing gravidity is not related to increased child-specific HLA antibody formation in secundigravidae with a prior miscarriage. Further details about the miscarried fetus itself or paternal HLA typing will be required to explain the observed different impact of a previous miscarriage and a previous successful pregnancy on child-specific HLA antibody formation during a subsequent successful pregnancy. These data may help to understand the mechanism of child-specific HLA antibody formation during a successful pregnancy that was preceded by a miscarriage and therefore will have implications in the transplantation field.

## Author Contributions

All the authors met the authorship criteria as described by *Frontiers in Immunology*. KG, GH, SS, and ES were involved in design of the work and interpretation of the data. HD and IH were involved in acquisition of the data. All the authors were involved in drafting or revising the manuscript and approved the final version. All the authors agreed to be accountable for all aspects of the work in ensuring that questions related to the accuracy or integrity of any part of the work are appropriately investigated and resolved.

## Conflict of Interest Statement

The authors of this manuscript have conflicts of interests to disclose. The UMCU has filed a patent application on the prediction of an alloimmune response against mismatched HLA. ES is listed as inventor on this patent. The other authors have no conflicts of interest to disclose as described by the *Frontiers in Immunology*.

## Abbreviations

IPA, inherited paternal HLA antigens; MFI, mean fluorescence intensity; PIRCHE-II, Predicted Indirectly ReCognizable HLA Epitopes presented by HLA class-II.
